# Transcriptome Analysis Reveals the Role of *OsCBM1* in Rice Defense Against *Xanthomonas oryzae pv.oryzae*

**DOI:** 10.3390/biom15020287

**Published:** 2025-02-14

**Authors:** Shuaijun Bie, Youlun Xiao, Li Zhang, Yong Liu, Xiaomin He, Jing Peng, Hongjun Xie, Yang Gao, Xiaojuan Li, Xinqiu Tan, Renyan Huang, Deyong Zhang

**Affiliations:** 1 College of Plant Protection, Hunan Agricultural University, Changsha 410128, China; bsj010208@outlook.com (S.B.); liuyong@hunaas.cn (Y.L.); 2 Hunan Plant Protection Institute, Hunan Academy of Agricultural Science, Changsha 410125, China; xiaoyoulun@hunaas.cn (Y.X.); pengjing@hunaas.cn (J.P.); tanxinqiu@hunaas.cn (X.T.); 3 Nuclear Agriculture and Space Breeding Research Institute, Hunan Academy of Agricultural Sciences, Changsha 410125, China; zhangliah@hunaas.cn; 4 College of Agriculture, Hunan Agricultural University, Changsha 410128, China; hexiaomin20201118@163.com; 5 Hunan Rice Research Institute, Hunan Academy of Agricultural Science, Changsha 410125, China; xhj1110@hunaas.cn; 6 Yuelushan Laboratory, Changsha 410125, China

**Keywords:** malectin-like domain-containing protein, *OsCBM1*, *Xanthomonas oryzae pv. oryzae*, reactive oxygen species, phytohormones, rice

## Abstract

Carbohydrate-binding malectin/malectin-like domain-containing proteins (CBMs) represent a newly discovered subclass of lectins that participate in various biological processes across the bacterial, animal, and plant kingdoms. The *OsCBM1* gene in rice enhances reactive oxygen species (ROS) burst, contributing to drought-stress tolerance. Nonetheless, the functions of *OsCBM1* in response to biotic stress remain poorly understood. In this research, we discovered that *OsCBM1* was activated by *Xoo* infection, and overexpression of *OsCBM1* increased rice resistance to bacterial blight, while suppression of its expression shows the opposite trend. *OsCBM1* may influence resistance to bacterial blight by regulating ROS burst and the SA signaling pathway through RNA-seq analysis. Overexpression of *OsCBM1* increased SA content and enhanced activities of SOD, POD, and CAT enzymes, whereas knockdown of *OsCBM1* exhibited the opposite trend. The expression of genes associated with the SA and enzyme activity pathways was validated through quantitative real-time polymerase chain reaction (qRT-PCR). These results further clarify the function of *OsCBM1* in biotic stress resistance, providing references for disease-resistant rice breeding.

## 1. Introduction

Carbohydrate-binding malectin/malectin-like domain-containing proteins (CBMs), as members of the lectin subfamily, play crucial roles in the biological processes of animals, plants, and microorganisms [1,2]. In plants, CBMs are malectin-like receptor kinases, a newly identified subfamily of receptor-like kinases (RLK). They are also referred to as *Catharanthus roseus* receptor-like kinase-like proteins (CrRLK1Ls) and play a role in plant immunity, cell wall completion, and other biological functions [3,4,5]. The CrRLK1L family of proteins in plants all possess a malectin-like structural domain, a transmembrane structural domain, and an intracellular serine/threonine kinase structural domain [6]. FERONIA (FER) is the most functional protein in the CrRLK1Ls family, interacting with ROS and Ca^2+^ to mediate pollen tube signaling in the reception and mechanical signaling of *Arabidopsis thaliana* [7,8]. The CBM1 in rice was discovered to contain just one malectin-like structural domain. and OsCBM1 forms a physical interaction with *OsRbohA* and *OsRacGEF1* to cooperatively regulate ROS production, thereby controlling the sensitivity to the abiotic stress of drought [9]. There are currently no reports on whether the *OsCBM1* gene in rice plays a role in regulating the plant’s response to environmental stress.

Rice bacterial blight, triggered by *Xanthomonas oryzae pv*. *oryzae* (*Xoo*), can threaten rice yields [10]. Throughout the process of natural selection, plants have evolved an inherent immune response to combat infestations from pathogenic bacteria. Receptor proteins on plant cell surfaces, known as pattern recognition receptors (PRRs), can detect pathogenic bacteria and activate immunity. This detection process is called pathogen-associated molecular pattern (PAMP) recognition. The immune response triggered by PRRs recognizing PAMPs is associated with what is known as PAMP-triggered immunity (PTI) [11]. In addition, pathogenic bacteria can secrete virulence factors that inhibit plant PTI, and these factors can infiltrate cells and induce plant disease. Plants have coping strategies against pathogens, recognizing specific virulence effectors to trigger a distinct immune response called Effector-Triggered Immunity (ETI), distinct from the Pathogen-Triggered Immunity (PTI) [12]. While PTI serves as the initial defense, reacting to widely conserved pathogen molecules, ETI targets specific pathogen proteins. Despite their differences, PTI and ETI share or intersect in their downstream signaling pathways [13].

Reactive oxygen species (ROS) are important signaling mechanisms that govern plant development and stress response [14,15]. ROS can be produced and accumulated rapidly during plant infection by pathogens [16]. Energy or electron transfer generates reactive oxygen species (ROS); typical examples include hydrogen peroxide (H_2_O_2_), superoxide radical anion (O_2_^−^), hydroxyl radicals (OH^−^), and singlet oxygen (^1^O_2_) [17]. ROS bursts are essential in rice’s response to various pathogen infections; for instance, the rice heavy metal transporter *OsNRAMP1* significantly contributes to plant immunity by managing the equilibrium of metal ions and ROS [18]. *OsATL32* ubiquitinates the *OsRac5*-*OsRbohB* module, which produces reactive oxygen species, thereby inhibiting rice immunity [19]. SPL50, an ARM repeat protein crucial for regulating ROS metabolism and boosting resistance to blast disease [20]. However, excessive levels of ROS in plants can cause damage to plants. To balance the outbreak of ROS, the enzymatic systems containing superoxide dismutase (SOD), catalase (CAT), and peroxidase (POD) play a crucial role. For example, the overexpression of *OsANN9* led to heightened activities of SOD, POD, and CAT, which helped to maintain reactive oxygen species homeostasis and improve drought resistance [21]. Similar to the antioxidant system, the phytohormonal signaling pathways play a significant role in enhancing host resistance by mediating downstream signal transduction and safeguarding cells from oxidative damage. Various phytohormones responsive to biotic stress, including salicylic acid (SA), ethylene (ET), jasmonic acid (JA), abscisic acid (ABA), melatonin, and strigolactone, are crucial for maintaining the equilibrium of redox processes within plant cells [14,15,22,23].

Here, we report that overexpression of *OsCBM1* enhanced resistance in rice to the bacterial blight, reducing the lesion length ratio by approximately 36.2% to 84.6% against different pathogens. While suppression of this gene exhibited the opposite trend. This result was due to altered SA content and activity of ROS-related enzymes revealed through RNA sequencing and assays of enzyme activity. Our research findings provide a theoretical basis and breeding reference for the involvement of *OsCBM1* in rice disease resistance.

## 2. Materials and Methods

### 2.1. Plant and Bacterial Material

Rice (*Oryza sativa* ssp. *Geng*) Nipponbare (Nip), *OsCBM1*-RNAi plants, and *OsCBM1*-OE plants were provided by the State Key Laboratory of Crop Stress Biology in Arid Area, College of Life Sciences, Northwest A&F University [9]. Seedlings were grown in the paddy field of Hunan Academy of Agricultural Sciences in Changsha under natural conditions. The study utilized *Xanthomonas oryzae pv.oryzae*, including two Philippine strains (PXO99, PXO341) and two Chinese strains (Zhe173 and Fuj23) provided by the National Key Laboratory of Crop Genetic Improvement, National Center of Plant Gene Research, Huazhong Agricultural University. All *Xoo* strains were cultured at 28 °C on nutrient agar medium.

### 2.2. Pathogen Inoculation

To assess the response of rice plants to *Xoo*, the plants were infected with the *Xoo* strains using the leaf clipping technique during the booting stage (panicle development) [24]. The disease was evaluated by measuring lesion length 14 days after inoculation. Each bacterial inoculation test was performed at least twice. Furthermore, the disease in some plants was assessed by analyzing bacterial proliferation counting colony-forming units as previously described [25]. To assess bacterial growth, a single leaf infected with *Xoo* from each plant was used as a single replicate, with three plants in total examined for each sample. The bacterial proliferation in rice leaves was assessed by counting colony-forming units [26].

### 2.3. Measurement of ROS-Related Enzyme Activities

Rice plants (Nipponbare (Nip), *OsCBM1*-RNAi, and *OsCBM1*-OE plants) were cultivated in the paddy field until the booting stage, and leaves were collected 12 h after inoculation with the bacterial blight pathogen and quickly frozen using liquid nitrogen. The activities of superoxide dismutase (SOD) and peroxidase (POD) were evaluated using assay kits following the manufacturer’s guidelines (Beijing Solarbio Science and Technology, Beijing, China). The catalase (CAT) activity was determined using assay kits according to the manufacturer’s guidelines (Beijing Boxbio Science and Technology, Beijing, China). All materials were tested in three technical replicates, with WT (Nipponbare) as the control.

### 2.4. RNA Extraction, cDNA Library Construction, and Sequencing

Rice leaves of Nipponbare (Nip), *OsCBM1*-RNAi, and *OsCBM1*-OE plants were collected in liquid nitrogen at 12 h after inoculation with *Xoo* at the booting stage. All materials were tested in three technical replicates, with WT (Nipponbare) as the control. Total RNA was extracted according to TRIzol reagent (Invitrogen, Carlsbad, CA, USA). The integrity of RNA was evaluated with an Agilent 2100 Bioanalyzer (Agilent Technologies, Santa Clara, CA, USA). RNA purity and quantity were verified using a NanoDrop 2000 spectrophotometer (Thermo Scientific, Waltham, MA, USA). Transcriptome sequencing and analysis were conducted by OE Biotech Co. (Shanghai, China), and transcriptome libraries were constructed using the VAHTS Universal V5 RNA-seq Library Preparation Kit. The libraries were sequenced using the Illumina Novaseq 6000 sequencing platform, and the raw data of this paper can be obtained from the GSE288740 database of NCBI.

### 2.5. DGEs Analysis and Functional Annotation

DEG analysis was conducted using DESeq2 software (version 1.12.3), with a log2 fold change greater than 1.5 and a false discovery rate (FDR) below 0.05 as the screening criteria to filter out the differential genes among the three groups, and the FDR was obtained by correcting for the significant *p*-value. Gene ontology (GO) functional annotation and Kyoto Encyclopedia of Genes and Genomes (KEGG) pathway enrichment analysis of the genes were performed using the analysis of the differentially expressed levels of the genes in the samples. FDR was obtained by correcting the significant *p*-value.

### 2.6. qRT-PCR Validation

Seven potential genes were chosen for qRT-PCR analysis to ensure confidence in the transcriptome data. Reverse transcription was performed using the 5X RT kit (abm (New York, NY, USA), Cat. No. G592); qRT-PCR was performed using the BlasTagTM 2X qPCR premix (abm, Cat. Nos. G891, G892), and the procedure was carried out as follows: The process began with 3 min at 95 °C, followed by 40 cycles of 95 °C for 15 s and 60 °C for 1 min each, and the *OsActin* gene was utilized as an internal control in all qRT-PCR. All samples were subjected to three independent replicates, and the results were analyzed using 2^−ΔΔCt^.

### 2.7. Statistical Analysis

Statistical analyses of enzyme activity and lesion length were performed using independent samples *t*-test and one-way variance (ANOVA) using SPSS version 27.0 software (SPSS, Chicago, IL, USA); *p* < 0.05 was considered significant.

## 3. Results

### 3.1. The Expression of OsCBM1 Was Triggered in Rice upon Pathogen Xoo Challenge

**Figure 1:**
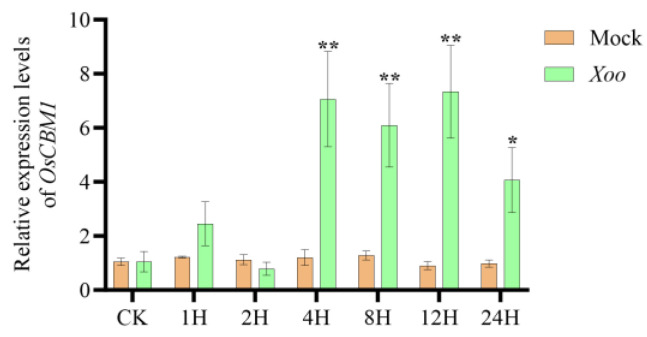
Relative transcription levels of rice *OsCBM1* after *Xoo* inoculation at 0 h, 1 h, 2 h, 4 h, 8 h, 12 h, and 24 h using qRT-PCR assay. Error bars indicate SE (* *p* < 0.05, ** *p* < 0.01).

To explore whether *OsCBM1* can effectively protect rice against *Xoo* infestation, we examined the expression level of *OsCBM1* in response to the pathogen *Xoo* inoculated at 0 h, 1 h, 2 h, 4 h, 8 h, 12 h, and 24 h using a qRT-PCR assay. and it was observed that the expression of *OsCBM1* began to increase significantly at 4 h post-inoculation and reached the highest expression at 12 h, with the highest induced expression exceeding 7-fold. (Figure 1). The results indicated that *OsCBM1* expression is induced in response to *Xoo* infestation in rice.

### 3.2. OsCBM1 Positively Regulated Rice Resistance to Xoo Infestation

**Figure 2:**
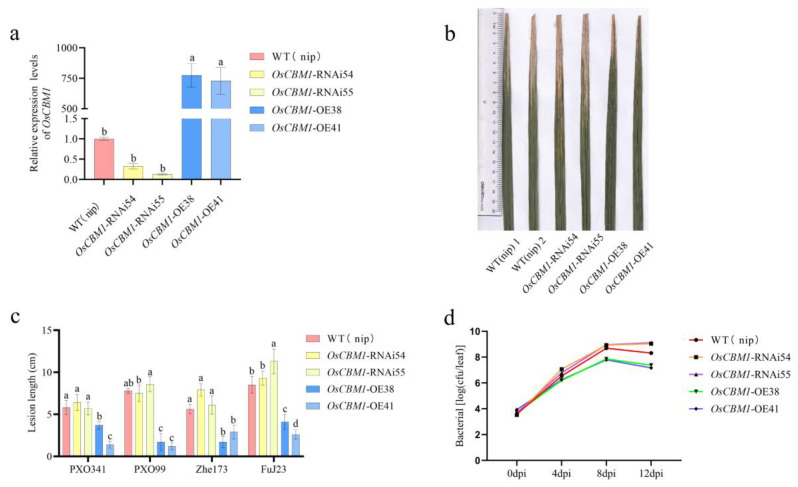
*OsCBM1* positively regulates the resistance to *Xoo.* (**a**) qRT-PCR analysis of the relative expression level of *OsCBM1* in WT, RNAi, and OE. (**b**) Measurements of infestation length of four *Xoo* strains inoculated with 14d pathogens from WT, OE, and RNAi plants. (**c**) Images of *Xoo* infestation of three strains with measurements of 14d pathogen spot length. (**d**) Growth graphs of the number of leaves collected from *Xoo*-infested OE, WT, and RNAi plants of three strains at 0 d, 4 d, 8 d, and 12 d plotted on medium after beating and dilution. Data from three biological replicates are shown as means ± SE (n = 3). Barcodes labeled with different letters represent significantly different (*p* < 0.05) values according to a one-way ANOVA to analyze the data.

To explore the functional role of *OsCBM1* in resistance to the disease caused by *Xoo*, we obtained knockdown lines and overexpression lines *OsCBM1*-RNAi54, *OsCBM1*-RNAi55, *OsCBM1*-OE38, and *OsCBM1*-OE41 for additional analysis. Expression levels of *OsCBM1* were markedly elevated in *OsCBM1*-OE plants and decreased in *OsCBM1*-RNAi plants (Figure 2a). We assessed the disease responses of *OsCBM1*-RNAi and *OsCBM1*-OE plants to *Xoo* by inoculation with *Xoo* during the booting stage. The *OsCBM1*-OE plants exhibited significantly enhanced resistance to various *Xoo* strains from the Philippines (PXO341, PXO99) and China (Zhe173, FuJ23), with lesion lengths approximately 2.7 to 6.6 cm shorter than the wild type and reduced *Xoo* growth rates (Figure 2b–d). However, all the *OsCBM1*-RNAi plants showed longer or similar lesion length and *Xoo* growth rates compared to wild type after inoculation with *Xoo* (Figure 2b–d). Taken together, our findings showed that *OsCBM1* was able to trigger plant immunity after *Xoo* infestation.

### 3.3. Transcriptome Analysis Identifies Genes Regulated by OsCBM1

**Figure 3:**
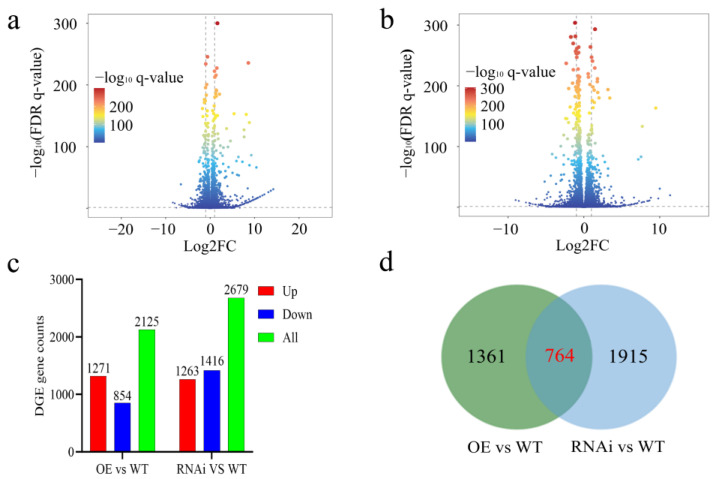
Transcriptomic analysis of *OsCBM1*-RNAi, *OsCBM1*-OE, and WT (Nip) after *Xoo* inoculation. (**a**) Differential gene changes in OE vs. WT. (**b**) Differential gene changes in RNAi vs. WT. (**c**) Number of up-regulated vs. down-regulated expression of differential gene changes in OE vs. WT vs. RNAi vs. WT. A total of 1317 genes up-regulated and 854 genes down-regulated expression in OE vs. WT; 1263 genes up-regulated and 1416 genes down-regulated expression in RNAi vs. WT. (**d**) Venn diagram analysis of DGEs between OsCBM1-OE vs. WT and OsCBM1-RNAi vs. WT. A total of 764 genes are shared, 1361 DGEs exist independently in OsCBM1-OE vs. WT, and 1915 DGEs exist independently in OsCBM1-RNAi vs. WT.

To gain a deeper understanding of the molecular mechanism through which *OsCBM1*-regulated genes enhance *Xoo* tolerance in rice, we conducted transcriptome analysis of *OsCBM1*-RNAi plants and *OsCBM1*-OE plants, as well as wild-type plants, after *Xoo* inoculation for 12 h. All materials were tested in three technical replicates, with WT (Nipponbare) as the control. Differentially expressed genes (DGEs) were identified (Figure 3a,b). Among the 2125 DEGs, 1217 genes showed increased expression, and 854 genes showed decreased expression between *OsCBM1*-OE and WT; there were more DGEs, with 1263 genes up-regulated and 1416 genes down-regulated, compared to *OsCBM1*-RNAi and WT (Figure 3c). Through Venn diagram analysis, it was found that 764 DEGs were differentially expressed in both OsCBM1 overexpression and suppression plants. Additionally, 1361 DEGs were differentially expressed only in OsCBM1 overexpression plants, while 1915 DEGs were differentially expressed in OsCBM1 suppression plants (Figure 3d).

### 3.4. Analysis of DGEs in GO

**Figure 4:**
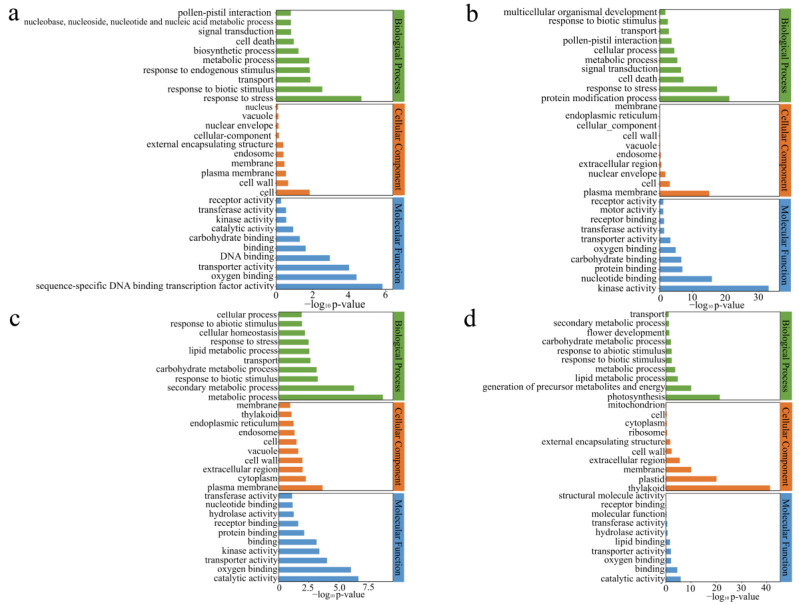
GO analysis of DGEs in WT, *OsCBM1*-RNAi, *OsCBM1*-OE. (**a**) GO enrichment analysis of *OsCBM1*-OE vs. WT up-regulated genes. (**b**) GO enrichment analysis of *OsCBM1*-RNAi vs. WT up-regulated genes. (**c**) GO enrichment analysis of *OsCBM1*-OE vs. WT down-regulated genes. (**d**) GO enrichment analysis of *OsCBM1*-RNAi vs. WT down-regulated genes.

To ascertain the potential functions of DEGs, we conducted GO enrichment analysis to investigate the pathways and biological processes involved with *OsCBM1*. The genes that were up-regulated in *OsCBM1*-OE vs. WT were most abundantly represented in the biological processes categories of response to stress and response to biotic stimulus. Other highly enriched categories included cell in the category of cell components and sequence-specific DNA binding transcription factor activity, oxygen binding, transporter activity, and DNA binding in the category of molecular function (Figure 4a). However, the GO terms for *OsCBM1*-RNAi vs. WT indicated that up-regulated genes were predominantly concentrated in protein modification processes, response to stress, plasma membrane, kinase activity, and nucleotide binding were significantly enriched (Figure 4b).

The most abundant terms for *OsCBM1*-OE vs. WT down-regulated genes were metabolic process and secondary metabolic process in the biological processes categories, and plasma membrane in the cell component category, as well as catalytic activity and oxygen binding in the molecular function category (Figure 4c). Compared with down-regulated genes in the *OsCBM1*-OE vs. WT, the *OsCBM1*-RNAi vs. WT had unique GO terms, including photosynthesis, generation of precursor metabolites and energy, thylakoid, and plastid (Figure 4d).

### 3.5. Analysis of DGEs in KEGG

**Figure 5:**
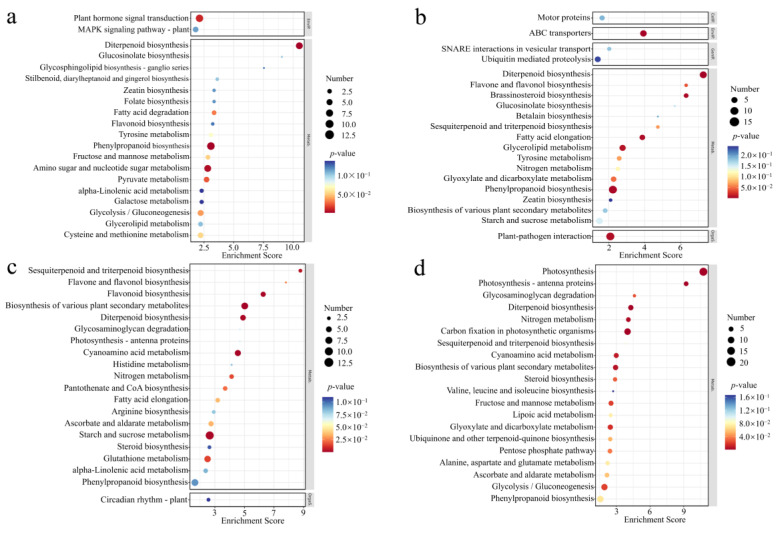
KEGG analysis of DGEs in WT, *OsCBM1*-RNAi, *OsCBM1*-OE. (**a**) KEGG analysis of *OsCBM1*-OE vs. WT up-regulated genes. (**b**) KEGG analysis of *OsCBM1*-RNAi vs. WT up-regulated genes. (**c**) KEGG analysis of *OsCBM1*-OE vs. WT down-regulated genes. (**d**) KEGG analysis of *OsCBM1*-RNAi vs. WT up-regulated genes.

Comparison of DGEs in *OsCBM1*-OE vs. WT and *OsCBM1*-RNAi vs. WT. *OsCBM1*-OE vs. WT up-regulated genes were primarily concentrated in amino sugar and nucleotide sugar metabolism, phenylpropanoid biosynthesis, and diterpenoid biosynthesis, as well as plant hormone signal transduction (Figure 5a). The pathways enrichment for *OsCBM1*-RNAi vs. WT up-regulated genes mainly included plant–pathogen interaction, phenylpropanoid biosynthesis and diterpenoid biosynthesis (Figure 5b). *OsCBM1*-OE vs. WT down-regulated genes were concentrated in several metabolic pathways, including starch and sucrose metabolism, biosynthesis of diverse plant secondary metabolites, phenylpropanoid biosynthesis, and glutathione metabolism (Figure 5c). The most differentially enriched pathways in *OsCBM1*-RNAi vs. WT down-regulated genes included photosynthesis, carbon fixation in photosynthetic organisms, phenylpropanoid biosynthesis, and glycolysis/gluconeogenesis (Figure 5d). These differences in pathway enrichment may result in altered disease resistance of *OsCBM1*-OE and *OsCBM1*-RNAi lines.

### 3.6. OsCBM1 Regulates the Resistance of Rice to Xoo Through the SA Pathway

**Figure 6:**
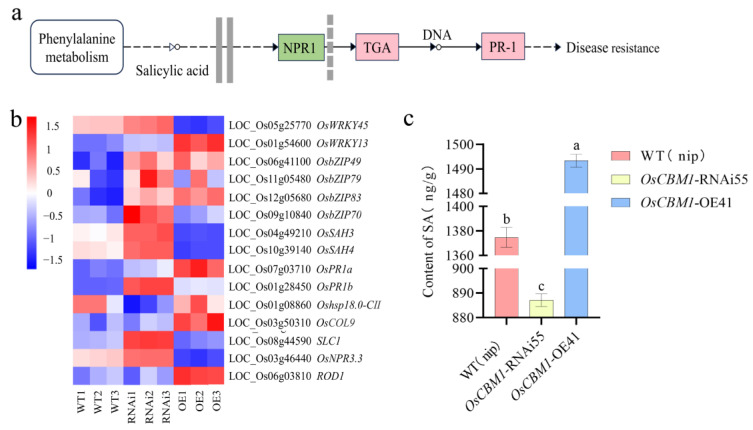
SA content assay and expression levels of DGEs in OE, WT, RNAi. (**a**) Involvement of SA in plant hormone signal transduction pathways for disease resistance SA. (**b**) Analysis of SA-related differential genes. The expression data in the heatmap are derived from changes in FPKM values of genes related to the SA signaling pathway. (**c**) Relative amount of SA in WT (nip), *OsCBM1*-RNAi55, *OsCBM1*-OE41. Data from three biological replicates are shown as mean ± SE (n = 3). Error bars indicate SE. Barcodes marked with different letters indicated significantly different (*p* < 0.05) values according to a one-way ANOVA to analyze the data.

To explore the downstream regulatory pathways of *OsCBM1* functions, we focused on the genes involved in the phytohormone signaling pathways. The SA-mediated disease resistance pathway has several DGEs affecting related pathways, such as NPR1, TGA transcription factors, and protein-related (PR) genes (Figure 6a). In addition, we screened for fifteen DGEs related to SA synthesis and metabolism, which collectively regulate SA-mediated immune processes (Figure 6b). To further verify whether the *OsCBM1* affects *Xoo* resistance by regulating the content of SA, we determined the levels of SA in rice after *Xoo* infestation for 12 h in *OsCBM1*-OE, *OsCBM1*-RNAi, and WT. It showed that the content of SA was importantly elevated in *OsCBM1*-OE plants compared to WT, while the opposite result was obtained for *OsCBM1*-RNAi plants (Figure 6c). These results suggest that the SA signaling pathway is essential for the disease resistance function of *OsCBM1.*

### 3.7. OsCBM1 Regulates the Resistance of Rice to Xoo Through ROS Burst

**Figure 7:**
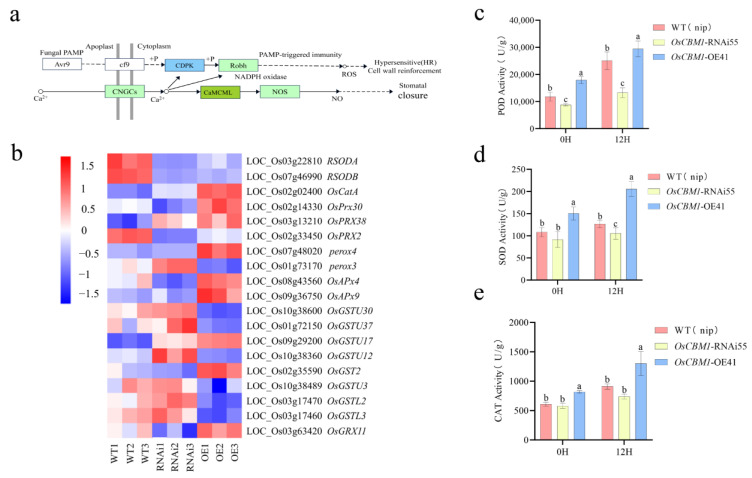
Determination of ROS-related enzyme activities and analysis of DGEs in three plants of OE, WT, and RNAi before and after *Xoo* infestation. (**a**) ROS-involved pathways of plant–pathogen interactions. (**b**) Heatmap analysis of ROS-related DGEs. The expression data in the heatmap are derived from changes in FPKM values of genes related to the ROS signaling pathway. (**c**) Determination of SOD enzyme activities. (**d**) Determination of POD enzyme activities. (**e**) Determination of CAT enzyme activities. Data from three biological replicates are shown as mean ± SE (n = 3). Error bars indicate SE. Barcodes labeled with different letters represented significantly different (*p* < 0.05) values according to a one-way ANOVA to analyze the data.

Within the plant–pathogen interaction pathway, ROS is crucial for inducing plant disease resistance. Phosphorylation of Ca^2+^-regulated CDPKs upstream of ROS in the plant–pathogen interactions pathway plays an important role (Figure 7a). In addition, we screened ROS synthesis, metabolic regulation, and related enzyme genes in DEGs, which also included glutathione peroxidase, glutathione sulfurtransferase, and intercellular redox balance (Figure 7b). *OsCBM1* participates in ROS production of plants with *OsCBM1*-OE plants containing higher levels of ROS (H_2_O_2_ and O^2−^) than *OsCBM1*-RNAi plants [9]. It showed that ROS burst could be crucial in conferring resistance against *Xoo* mediated by *OsCBM1*. Elevated ROS levels were also accompanied by elevated activities of related scavenging enzymes. Therefore, we analyzed the concentration of SOD (superoxide dismutase), POD (peroxidase), and CAT (catalase) in leaves of *OsCBM1*-RNAi plants and *OsCBM1*-OE plants together with wild type. Both relatively higher SOD, POD, and CAT concentrations in *OsCBM1*-OE plants than in the wild type, irrespective of whether they are inoculated with *Xoo* or not, while the trend is reversed in *OsCBM1*-RNAi plants (Figure 7c–e). These findings indicate that the increasing enzymatic activities of SOD, POD, and CAT could be involved in regulating the ROS burst in *OsCBM1*-OE plants, resulting in enhanced resistance against *Xoo* infection.

### 3.8. qRT-PCR Analysis of Related Genes

**Figure 8:**
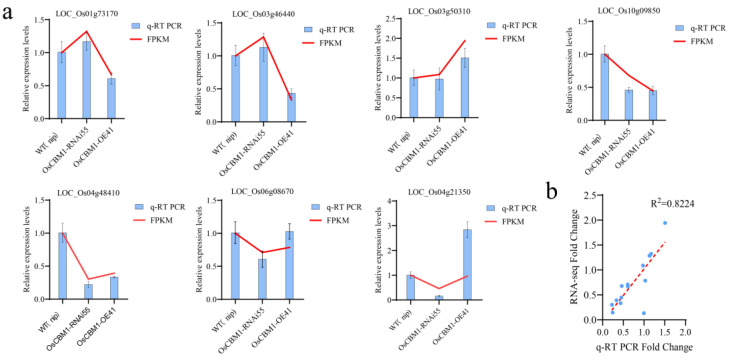
qRT-PCR and RNA-seq results consistency validation. (**a**) Seven DEGs associated with ROS and SA were selected for qRT-PCR validation. Error bars indicate SE. (**b**) Validation of the consistency of RNA-seq and qRT-PCR results correlation; blue dots show the correlation between RNA-seq of DGEs and qRT-PCR fold change ratio, and the red dotted line indicates the correlation coefficient with an R^2^ of 0.8224.

To verify the accuracy of RNA-seq, several ROS and SA-related genes were chosen for qRT-PCR analysis. Overall, the gene expression results from the qRT-PCR aligned with the RNA-seq findings, suggesting a comparable pattern between the qRT-PCR and transcriptome data. (Figure 8a). The relationship among the fold change in qRT-PCR and RNA-seq (FPKM) data for the seven differentially expressed genes (DEGs) was analyzed using Pearson correlation and linear regression, revealing a notably positive correlation (Figure 8b).

## 4. Discussion

### 4.1. OsCBM1 Enhances Rice Resistance to Bacterial Blight

In a previous study, *OsCBM1* was validated to be solely a malectin-like structural domain [9]; CBMs in plants are *Catharanthus roseus* receptor-like kinase-like proteins (CrRLK1Ls), which are also part of a subclass within the receptor-like kinase (RLK) family. Every protein in this family exhibits a malectin-like structural domain and is involved in cell wall integrity, plant immunity, and other processes [4,6,7]. The transcript of *OsCBM1* is primarily expressed in leaves, and the gene is localized to the endoplasmic reticulum and plasma membrane. It can be stimulated for expression by a broad spectrum of biotic hormones and abiotic stress factors. These features suggest that *OsCBM1* is a stress-responsive protein [9]. In this study, *OsCBM1* positively controls the resistance of rice to bacterial blight. Firstly, *OsCBM1* showed increased expression against *Xoo* infection (Figure 1). Secondly, the *OsCBM1*-OE plants exhibited markedly enhanced resistance to different *Xoo* strains from the Philippines (PXO341, PXO99) and China (Zhe173, FuJ23), whereas the *OsCBM1*-RNAi plants showed significantly reduced resistance (Figure 2b,c). Third, the growth rate of bacterial blight in *OsCBM1*-OE plants showed a marked reduction compared to the wild type (Figure 2d).

### 4.2. ROS Bursts Are Essential in the Positive Regulation of Rice Bacterial Blight Resistance by OsCBM1 

ROS possess multiple antimicrobial functions in plants, including directly killing pathogens, triggering phytochelatin synthesis, inducing programmed cell death, and activating defense genes [16,27,28]. However, excessive ROS can be toxic to cells, so plants have innately evolved to maintain ROS balance through enzymatic and non-enzymatic scavengers. Enzymatic scavengers like SOD, POD, CAT, APX, and others modulate ROS homeostasis [16]. Here, we believe that *OsCBM1* may confer resistance by regulating the burst of ROS. First, the differentially expressed genes from the transcriptome are enriched in ROS-related pathways through KEGG pathway analysis (Figure 4). Some genes in the ROS-related pathways are validated by qRT-PCR, which is consistent with the transcriptomic data (Figure 8). Third, through enzymatic activity assays, it was found that the *OsCBM1* gene positively regulates the enzymatic activity changes in SOD, POD, and CAT both before and after inoculation with *Xoo* (Figure 7). Furthermore, Rbohs are crucial for plant stress tolerance. During rice infestation with *M. oryza*, *OsRbohA* and *OsRbohB* in rice are activated for expression [29]; *OsCBM1* directly engages with *OsRbohA*, enhancing ROS levels and improving drought tolerance in rice [9]. *OsRacGEF1* was identified as a ROP nucleotide exclusive to plants exchange factor (PRONE)-type GDP/GTP exchange factor (GEF) family gene. It is activated by chitin at the plasma membrane, engages with *OsCERK1*, and has its S549 phosphorylated by *OsCERK1*. Together, they co-regulate resistance to rice blast disease [30]. *OsCBM1* physically interacts with OsRacGEF1 to positively regulate ROS production [9]. These results demonstrate that *OsCBM1* may promote the burst of ROS and thereby boost rice defense against bacterial blight through interaction with *OsRacGEF1* and *OsRbohA*.

### 4.3. OsCBM1 May Regulate Rice Resistance to Bacterial Blight by Increasing Salicylic Acid (SA) Content

Salicylic acid (SA) is regarded as a vital hormone for plant defense, enhancing resistance to biotrophic and semibiotrophic pathogens. It is integral to the fundamental defense mechanisms, the intensification of localized immune reactions, and the development of systemic acquired resistance [31]. In this study, we propose that *OsCBM1* confers disease resistance because overexpression of this gene increases the content of SA. The reason is that through transcriptomic KEGG enrichment of hormone signaling pathways, variation in the expression of SA-associated genes and changes in SA content were confirmed by qRT-PCR and hormone assays (Figure 4, Figure 7 and Figure 8). SA mediates oxidase reductase activity to regulate wheat immunity against glume blight (*Septoria nodorum*) [32]. It also promotes resistance against *Pseudomonas syringae* in *Arabidopsis thaliana* [33]. Furthermore, SA is involved in regulating colonization of the root by particular bacterial families to mediate plant pathogen resistance [34]. The NPR1 protein plays a crucial role within the SA signaling cascade, and the level of NPR1 expression can influence the expression level of downstream PR-1 [35]. The differential expression of NPR1-related genes in *OsCBM1*-OE plants in this study may represent a pathway through which *OsCBM1* is involved in regulating SA-mediated disease resistance.

### 4.4. The Immunity of Rice Against Bacterial Blight Potentially Regulated by OsCBM1 Might Also Involve Other Mechanisms

In this study, we found that in the disease-resistant *OsCBM1*-OE plants, some disease-related genes are expressed, for example, *OsPR1a*, *OsWRKY71*, *ROD1*, *OsCYP71Z2*, and *OsPep3* (Figure S1). Quick activation of the *OsPR1a* protein is crucial for *Xa21*-dependent defense against *Xoo* [36]. *OsWRKY71* is crucial for the defense mechanism in rice. Elevating the expression of the *OsWRKY71* gene boosts rice resistance to virulent leaf blight by stimulating *OsNPR1* and *OsPR1b* at an earlier stage in the defense signaling pathway [37]. *OsPep3* enhances rice’s resistance to *Xoo*. Hormonal and transcriptomic analyses of *OsPep3*-treated leaves showed that JA biosynthesis, lipid metabolism, and phenylpropanoid metabolism may contribute to immunity triggered by *OsPep3* [38]. *OsCYP71Z2* can enhance rice resistance to pathogenic bacteria by regulating the synthesis of plant antitoxins, thereby [39]. Here, there are also some genes that are down-regulated in disease-resistant plants, such as *NRR*, *OsGLIP1*, *OsBON3*, *OsCaML2*, *RH1*, *RH2*, *RH3*, *OsMPK17, OsWRKY76,* and others, NRR acts as a negative regulator of disease resistance in rice and interacts with NH1 in rice. Overexpressing NRR plants showed increased sensitivity to *Xoo* [40]. *OsGLIP1* and *OsGLIP2* are lipases involved in the hydrolysis of lipids and adversely affect the defense response of rice against bacteria and fungi [41]. *OsBON3* promotes rice growth but negatively regulates leaf blight resistance. Pathogen infestation altered the subcellular localization of *OsBON1* and *OsBON3*, which could represent an initial reaction to pathogen invasion [42]. *OsCaML2*, a target of Osa-miR1422, adversely affects rice resistance to *Xoo*, and the microRNA *Osa-miR1422* improves rice disease resistance by suppressing *OsCaML2* [43]. *RH1*, *RH2,* and *RH3* are three NRR homologous proteins. *RH1*, *RH2,* and *RH3* can effectively inhibit NH1-mediated transcriptional activation and suppress natural immunity [44]. *OsMPK17* negatively regulates *Xa21*-dependent defense against *Xoo* in rice [45]. Overexpression of *OsWRKY76* reduces defense against leaf blight in rice [46].

## 5. Conclusions

Our results suggest the possibility that *OsCBM1* positively regulates rice defense against *Xoo* through altering SA levels and enzyme activities of SOD, POD, and CAT. Among them, *OsCBM1* is up-regulated following *Xoo* infection, and its overexpression boosts defense against bacterial blight in rice, whereas knockdown *OsCBM1* leads to decreased resistance. *OsCBM1* likely affects the plant’s defense by regulating the ROS burst and SA signaling pathways, as indicated by RNA-seq data. Enhanced expression of *OsCBM1* raises SA levels and enhances the activity of enzymes like eSOD, POD, and CAT, while reduced *OsCBM1* expression results in reduced enzyme activities. Gene expressions linked to SA and enzyme activity were confirmed using qRT-PCR. These results further clarify the function of *OsCBM1* in enhancing biotic stress resistance and offer insights for developing disease-resistant rice strains.

## Data Availability

All data generated are included in this article and its Supplementary Materials. The raw RNA-Seq data supporting the conclusions of this article are available in the GSE288740 database of NCBI.

## Supplementary Materials

The following supporting information can be downloaded at: https://www.mdpi.com/article/10.3390/biom15020287/s1, Supplementary Figure S1, heatmap analysis of resistance DGEs in OsCBM1-OE vs WT; Supplementary S1, primer sequence.
